# Nanoliposome-loaded antifungal drugs for dermal administration: A review

**DOI:** 10.18502/cmm.7.1.6247

**Published:** 2021-03

**Authors:** Peyman Asadi, Ahmad Mehravaran, Nahid Soltanloo, Mahdi Abastabar, Javad Akhtari

**Affiliations:** 1 Student Research Committee, Department of Medical Nanotechnology, School of Advanced Technologies in Medicine, Mazandaran University of Medical Sciences, Sari, Iran; 2 Infectious Diseases and Tropical Medicine Research Center, Resistant Tuberculosis Institute, Zahedan University of Medical Sciences, Zahedan, Iran; 3 Invasive Fungi Research Center/Department of Medical mycology, School of Medicine, Mazandaran University of Medical Sciences, Sari, Iran; 4 Department of Medical Nanotechnology, School of Advanced Technologies in Medicine, Mazandaran University of Medical Sciences, Sari, Iran; 5 Toxoplasmosis Research Center, Communicable Diseases Institute, School of Medicine, Mazandaran University of Medical Sciences, Sari, Iran

**Keywords:** Antifungal, Drug delivery, Liposome, Nanoparticle, Skin

## Abstract

Cutaneous fungal infections are the fourth most common health problem, which involves approximately one billion people worldwide. Drug delivery to the skin seems to be the best choice for superficial fungal infections. Topical formulations can release a sufficient amount of drug in therapeutical concentrations and permeate higher layers of the skin like the stratum corneum. As the outermost layer of the epidermis, the stratum corneum prevents the drug from penetrating the skin. Liposomes, especially nanosized as topical drug delivery systems to the skin, can show various functions depending on their size, lipids and cholesterol components, the percent of ingredients, lamellarity, and surface charge. Nanoliposomes can increase permeation through the stratum corneum, decrease systemic effects with their localizing actions, and overcome many dermal drug delivery obstacles. Antifungal drugs, such as croconazole, econazole, fluconazole, ketoconazole, terbinafine hydrochloride, tolnaftate, and miconazole entrapped in liposomes have indicated improved skin penetration and localizing effects. According to the literature review summarized in this paper, many studies have identified liposomes as a powerful carrier for topical antifungal drug delivery to the skin. However, a few studies introduced new generations of liposomes like ethosomes and transfersomes. This paper was conducted on almost all liposomal studies of antifungal drugs with dermal application.

## Introduction

According to a recent Global Burden of Disease report, cutaneous fungal infections are the fourth most common health problems which involve approximately one billion people worldwide [ 1 , 2 ]. Fungal skin infections are substantially divided into three categories, namely dermatophytoses, pityriasis versicolor, and cutaneous candidiasis [ 3 , 4 ]. Amongst these classifications, dermatophytes are the leading cause of superficial infections. It should also be noted that any skin surface can be prone to infection [ 4 - 6 ]. 

Dermatophytes usually cannot penetrate deeper layers of the skin, and their infection is limited to the epidermis [ 4 , 5 ]. There are seven genera of dermatophytes (i.e., *Trichophyton*, *Microsporum*, *Epidermophyton*, *Arthroderma*, *Nannizzia*, *Lophophyton*, and *Paraphyton*) [ 3 , 4 , 7 - 10 ]. Some fungal infections can spread throughout the world and affect up to 70% of the adult population [ 11 ]. In the USA, from 1979 to 2000, infections by fungal organisms increased by 207% which is the most significant increase, compared to other organisms [ 12 ].

Skin infections are the most common type of fungal infections, and the prevalence of superficial fungal infections has grown over the past two decades [ 5 ]. Recently, there has been an interest in the investigations on the development of new procedures for the better and more convenient treatment of patients with fungal infections. Drug delivery to the skin seems to be the best choice for superficial fungal diseases. The epidermis is the target place of pathogens for accumulation and proliferation. The treatment should include proper topical formulations that can release a sufficient amount of drug in therapeutic concentrations and permeate through higher layers of the skin, like the stratum corneum, to achieve suitable treatment [ 10 , 13 - 15 ]. Recently, nanomedicine, by combining nanote-chnology, pharmaceutical science, and biomedicals with the application of nanoparticles, has shown a lot of abilities in the field of drug delivery [ 16 - 18 ]. In the world of nano, with the interaction of nanocarriers in the sub-atomic states of skin layers, many efficacies are exhibited, such as overcoming the barriers [ 16 ]. Liposomes, as the first approved nano-drug delivery system by FDA, have shown great efficacy due to their versatility, safety, biocompatibility, targeted delivery, high capacity, and capability to deliver both hydrophobic and hydrophilic molecules [ 19 - 24 ]. For more than 60 years, these lipidic bilayers have been analyzed from multiple aspects and have shown stupendous advantages in many fields of medicine and pharmaceuticals as delivery systems. They have provided suitable circumstances for overcoming drug delivery deficiencies [ 22 , 23 , 25 , 26 ].

Due to the lack of efficacy of the existing drugs, lengthy treatments, and deficient recovery, the treatment of topical infections has always been a challenge [ 16 ]. For superficial fungal diseases, topical drug delivery systems seem to be appropriate and able to fix the shortcomings. Topical drug delivery systems have significant advantages over the other routes, such as lack of hepatic first-pass metabolism and gastrointestinal tract tribulation. 

Despite the versatility of this administration route, many difficulties, barriers, and complexities still existed in the treated skin [ 27 - 29 ]. The stratum corneum, as the outermost layer of the epidermis, is one of these barriers which prevents the drug from penetrating the underlying layers. Obviously, for the provision of an effective therapeutic outcome, drugs must cross this barrier [ 27 , 30 ].

Liposomes are small multi/unilamellar vesicles of pure phospholipids and cholesterol that have many drug delivery applications. Usage of liposomes, especially nanosized, as topical drug delivery systems to the skin can display various functions depending on their size, components, lamellarity, and surface charge [ 28 ]. Nanoliposomes can increase permeation through the stratum corneum [ 7 , 8 , 10 , 13 , 31 ], decrease systemic effects with their localizing actions, and overcome many obstacles of dermal drug delivery [ 8 , 26 , 27 ]. Dermal delivery means delivering drugs into a specific location within the skin to improve the local effects of drugs without systemic effects [ 32 - 34 ]. 

**Figure 1:**
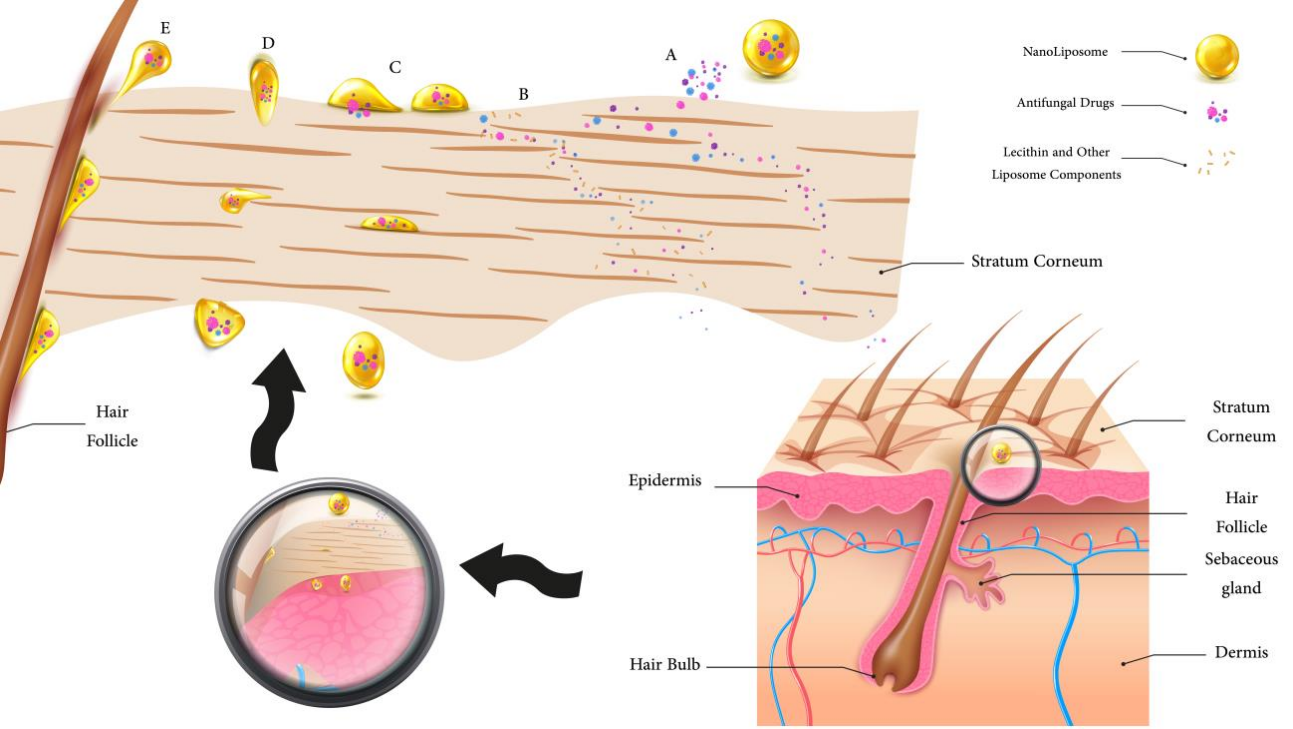
Summary of several possible mechanisms of liposome-skin interactions. A) Free drugs that release from liposomes on the surface of the skin, B) enhancers, like lecithin and other liposomes components, that enhance liposomes penetration, C) adsorption or fusion of liposomes by stratum corneum, D) penetration of intact liposomes to the skin, and E) trans appendageal penetration [ 30, 38 ]. This figure was designed by Adobe Illustrator software (version 2020) with modification of some elements that were "Designed by macrovector- Upklyak- brgfx/Freepik"

In the first study about the application of liposome on the skin as drug delivery systems published by Mezei and Gulasekharam in 1980, localizing effects of drug agents and more skin deposition were investigated [ 35 ]. Another experiment with identical methods and components was conducted in 1982 and had the same results [ 36 ]. These experiments were initial steps towards the suggestion of liposomes as drug delivery systems to the skin [ 37 , 38 ]. After these initial studies, some experiments introduced liposomes as promising carriers for topical uses on the skin by examining liposome-skin interactions from different aspects [ 7 , 39 , 40 ]. For liposome-skin interactions, several possible mechanisms are postulated that are summarized in Figure 1.

Antifungal drugs entrapped in liposomes have indicated improved skin penetration and localizing effects. This system also decreases percutaneous absorption [ 8 , 34 , 41 ], and since many liposomes consist of natural lipids and cholesterol, they do not show immunogenicity [ 42 ].

**Figure 2:**
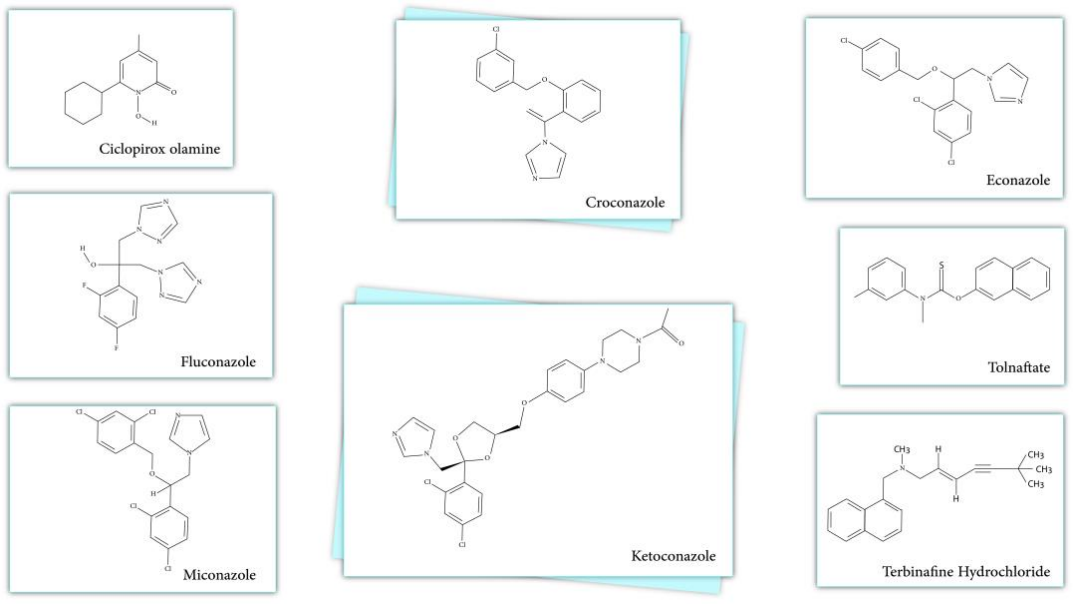
Structure of common antifungal drugs. This figure was designed by Adobe Illustrator software (version 2020)

Liposomal drug delivery systems can overcome drug resistance resulted from ineffective drugs administered to the site of infection. The present review was conducted to provide a better understanding of this area of research. At first, all of the studies in the field of topical drug delivery of antifungal agents by liposomes were collected from reliable sources and assessed. For the purposes of the study, a dataset of the drugs that were delivered was collected and classified in alphabetical order (Figure 2). Table 1 provides an overview of the collected data.

**Table1:** Overall overview of the collected data

Drug	Fungus	Liposome Ingredient	Size (nm)	Zeta potential (mV)	Reference
Ciclopirox	None	Phosphatidylcholine, Cholesterol	307- 844	-10.2 to -23.3	[ 44 ]
Croconazole	*Aspergillus flavus*, *Candida albicans*, *Chrysosporium tropicum* (keratinophyte), *Penicillium chrysogenum* and *Trichophyton rubrum* (dermatophyte)	Egg phosphatidylcholine	600 ( multilamellar vesicles) 200 ( small unilamellar vesicles)	Not Reported	[ 32 ]
Econazole	**Candida albicans**	Not Reported	105-555	Not Reported	[ 45 ]
Econazole	**Trichophyton rubrum**	Lecithin, Cholesterol (Pevaryl®-Lipoge)	Not Reported	Not Reported	[ 46 ]
Econazole	None	Pevaryl® cream	160-200	Not Reported	[ 47 ]
Fluconazole	*Candida albicans*	L-α-EggPhosphatidylcholine, Cholesterol	348	Not Reported	[ 49 ]
Fluconazole	None	Saturated Soy Lecithin (Phospholipon 90H), Cholesterol	3000-7300	-54.1 - -63.6	[ 50 ]
Fluconazole	None	1,2-Dipalmitoyl-sn-glycero-3-phosphocholine	37-72	+3 to +10	[ 28 ]
Ketoconazole	*Aspergillus niger*, *Candida tropicalis*	Soya Lecithin, Cholesterol	141.6	-45 mV	[ 54 ]
Ketoconazole	*Candida albicans*	Soybean Phosphatidylcholine	150	-27.9	[ 55 ]
Terbinafine Hydrochloride	None	Phospholipon 90H (Hydrogenated phosphatidylcholine), Dimyristoylglycero-3-phosphocholine (DMPC)	206.9-344.8	-25 to -37	[ 59 ]
Tolnaftate	*Candida albicans*	Soya lecithin, Cholesterol	119-284	Not Reported	[ 61 ]

### Ciclopirox olamine

Ciclopirox, known as one of the synthetic derivatives of hydroxypyridone, is an antifungal drug that is widely used for topical treatment. Mechanism of action of ciclopirox is to inhibit the transportation of electrons in mitochondria by inhibiting the essential enzymes involved in this process [ 27 , 43 ].

In one study, this drug was loaded into liposomes in various gel systems to supply controlled release of topical formulation for antifungal purposes. Various liposome formulations were prepared using thin-film hydration. Various liposome formulations were produced by a sufficient amount of phospha-tidylcholine and cholesterol and characterized based on different aspects, like encapsulation efficiency, drug loading, morphological character, surface charge, size analysis, and transmission electron microscopy results. 

In order to prepare topical gels containing liposomes, various polymers were used, such as agar (0.5% w/v), carrageenan (0.5% w/v) and carrageenan/agar in 1:1 ratio. These systems were found to have good size and stability characteristics. Drug release from the liposome single polymer gels and blend of polymers was much lower in comparison with liposomal suspensions [ 44 ]. 

### Croconazole

Croconazole is a synthetic drug with a tremendous topical antifungal activity that is derived from imidazole. Croconazole has a low molecular weight and its water solubility is very poor. Badry et al. in 2014 compared topical application potential of liposomal croconazole gel (LCG) and croconazole microemulsion gel (CMG). In their study, conventional gels were created by a few polymers, like chitosan, poloxamer 407, carbopol 971P, and sodium carboxymethyl cellulose. Moreover, the liposomes were prepared by egg phosphatidylcholine using the thin film method. Furthermore, they examined the *in vitro* release by cellophane membrane and all the formulations were applied to rat skin. 

According to their results, LCG and CMG can be very useful as croconazole carriers in topical delivery systems for the treatment of fungal diseases. Various CMGs and LCGs have a great performance against different fungi spp. Regarding the above-mentioned research, it is necessary to mention that micro-emulsion gel formulations showed better performance than other formulations in both antifungal activity and skin penetration studies [ 32 ].

EconazoleEconazole (ECZ) is one of the imidazole derivatives [ 29 ] which was synthesized for the first time in 1969. Its mechanism is similar to other azoles that inhibit 14-α demethylase enzyme related to ergosterol synthesis [ 43 ].

In one study, two commercially available formulations of ECZ (i.e., ECZ nitrate cream and ECZ liposome gel) were assessed with electron and light microscopy in terms of their effects on the uninfected human epidermis (reconstructed) and cutaneous candidosis human model. It was found that cream formulation had more harmful effects on both the uninfected human epidermis and cutaneous candidosis human model, compared to liposomal gel formulation. Besides, the liposomes containing ECZ showed effective antifungal activity without the side effects of ECZ cream formulation. Moreover, a great affinity was observed between liposomal preparation and *Candida albicans* [ 45 ].

Korting et al. in 1997 performed a controlled double-blind trial to compare the healing effects of ECZ liposomal gel (1%), branded ECZ cream (1%), and generic clotrimazole cream (1%) on patients with tinea pedis. This trial was performed on 535 patients within the age range of 18-65 years. All the prepared formulations were administered once daily for 14 days. The results indicated a higher treatment rate for ECZ liposomal gel, compared to other formulations. Besides, liposomal gels of ECZ showed a slightly higher level of tolerability [ 46 ].

A comparative study was conducted to evaluate different carrier systems as vehicles for antifungal drugs whose primary purpose was to develop aqueous micelle solutions for encapsulated clotrimazole, ECZ nitrate, and fluconazole (FLZ) as three antifungal drugs, compared to a commercial liposomal product. Characterization studies were performed on nanoparticles to assess their size distribution, zeta potential, and drug loading. The formulations were evaluated on human and porcine skin for the study of skin transport. 

Fluorescein was added to micelles, and the nanoparticles were analyzed by confocal laser scanning microscopy to determine the penetration routes and distributions of micelles in the skin. The loading efficiency of clotrimazole (as the most hydrophobic drug, compared to other azoles in the aforementioned experiment) was 20%, which was the lowest among others. Moreover, the loading efficacy of ECZ in MPEG-dihexPLA micelles was 98.3%, and its delivery was compared to the liposomal formulation available in the market (Pevaryl® cream (1% w/w ECZ)) for topical application. 

In the afore-mentioned study, six h after the application of ECZ MPEG-dihexPLA micelles on the porcine skin, the results showed a 13-fold higher deposition than the commercial liposomal formulation. Similarly, the results for human skin showed a 7.5-fold delivery improvement. These micellar systems showed higher efficacy of ECZ in delivery to the skin, compared to liposomal formulation (Pevaryl®) [ 47 ].

### Fluconazole

The FLZ is known as a water-soluble synthetic antifungal drug derived from triazole. This drug has been used commercially in the form of tablets and injections. It has many adverse side effects, like skin inflammation that limited its applications [ 48 , 49 ]. 

A study was conducted to overcome the defects of conventional FLZ treatments of skin infections and achieve suitable localized delivery to the skin. In the aforementioned research, liposomes and niosomes were produced as vesicular systems. The sizes of liposomes and niosomes were 348 nm and 326 nm, respectively. The fungicidal activity of this system was assessed on an albino rat with cutaneous candidiasis. 

In the above-mentioned study, the liposomal gel in both *in vitro* and *in vivo* experiments exhibited superior depositions of the drug into the skin. Furthermore, the fungicidal activity of liposomal FLZ showed great potential for topical delivery. In conclusion, liposomal FLZ was introduced as the most promising carrier for topical delivery of antifungal agents [ 49 ].

In another study, liposomal FLZ gel was compared with a non-liposomal formulation. In the aforemen-tioned experiment, liposomes were prepared by the thin-film hydration method, and their characterizations were assessed from several aspects. Drug deposition and permeation of the FLZ were examined on a model of rat skin. Skin permeation and the remaining of the drug in liposomal formulation indicated better efficacy of the gel, compared to the control group. As a result, the FLZ entrapped in liposomes was suggested as efficient for topical drug delivery to the skin for antifungal administration [ 50 ].

Topical delivery of FLZ as an antifungal agent by liposomes and ethosomes to treat fungal skin infections was assessed in a review article. At the end of the review, liposomes and ethosomes incorporated in gel formulation were advised for the delivery of FLZ for fungal skin infections [ 51 ].

Schwarz et al. in 2011 examined lysine derivatives for optimizing the liposomal structure and observing its effects on the skin permeation of FLZ as a fungicidal drug in 1,2-dipalmitoyl-sn-glycero-3-phosphocholine (DPPC) vesicles. The unilamellar liposomes were produced and characterized. The results showed a decrease in DPPC liposome size using lysine derivatives and retarding effect on skin permeation of FLZ. The above-mentioned study introduced these liposomes as promising systems for the percutaneous delivery of drugs [ 28 ].

### Ketoconazole

Ketoconazole (KTZ) is a drug that tackles fungal infections with an imidazole structure. The water solubility of this molecule is approximately 0.017 mg mL−1 at 25 °C. This drug is administered both topically and orally [ 52 ]. 

In one study, KTZ was entrapped into liposomes and prepared by the thin-film hydration method to be used for topical administrations. It is noteworthy that the main components of the formulation were soybean lecithin and cholesterol. In the afore-mentioned study, drug retention, *in vitro* drug release into the skin, and *in vitro* antifungal activity were examined, and the results indicated successful preparation. 

Afterward, Wistar albino skin was used to carry out a comparative study on *in vitro* KTZ permeation of a liposomal gel, plain drug gel, and plain drug cream (2% w/w). The liposomal gel demonstrated great efficacy, compared to the non-liposomal formulations. The KTZ release from liposomes was about 34% after 12 h, and the collected data from skin retention analysis showed superior drug retention. According to the results of the above-mentioned study, liposomal KTZ gel had the most antifungal activity, compared to other formulations [ 53 ].

The KTZ and neem (i.e., *Azadirachta indica*) extract incorporated in the liposomal gel system were developed to overcome seborrheic dermatitis. It should be noted that the principal method of preparation was thin-film hydration. Various characterization studies were performed to determine the zeta potential, particle size, drug release percentage, and entrapment efficiency.

Next, the antifungal potential was evaluated on *Candida tropicalis* and *Aspergillus niger*. Values of the distribution of the particle size, zeta potential, and entrapment efficiency were 141.6 nm, -45 mV, and about 88.9%, respectively. It is noteworthy that the formulations were also stable in refrigerated conditions. The results of the aforementioned research indicated the synergetic effect and great potential of KTZ and neem extract liposomal gel on topical treatment of seborrheic dermatitis [ 54 ].

Another study described novel lipid nanocarriers as topical delivery agents to enhance KTZ skin targeting. Ethosomes, conventional liposomes, deformable liposomes, and deformable liposomes containing ethanol were produced to deliver KTZ. At first, manufactured vesicles were characterized in terms of their particle size, zeta potential, and entrapment efficiency. Afterward, transmission electron microscopy (TEM) and confocal microscopy were performed. 

Porcine skin was used for the conduction of *in vitro* permeation experiment. Moreover, an *in vivo* experiment for KTZ accumulation was carried out on rat skin. The skin penetration of vesicles, that were labeled fluorescently, was evaluated by confocal microscopy. Sizes of all the vesicles were less than 160 nm with PDI < 0.3. Deformable liposomes containing ethanol showed remarkably enhanced skin deposition, compared to conventional liposomes (in both *in vivo* and *in vitro* experiments). Furthermore, the antifungal activity of deformable liposomes containing ethanol against *Candida albicans* showed an improvement. Finally, the aforementioned study introduced deformable liposomes containing ethanol as skin targeting enhancer for KTZ delivery in local therapy of antifungal diseases [ 55 ].

### Miconazole

Miconazole (MCZ) nitrate, an fungicidal agent, is one of the imidazole derivatives. Its topical application against fungal infections is not efficient due to a lack of exposure to deeper layers of skin. An experiment was conducted to overcome this defect by entrapping MCZ into liposomes as carriers and assessing various parameters involved in the function of liposomes. In the aforementioned study, the amount of MCZ, which was loaded into liposomes, ranged from 7.2 mg per 125 mg to 9.76 mg per 130 mg of total lipid.

Results of the *in vitro* permeation and retention in skin indicated the great potential of liposomes as topical drug carriers, compared to conventional formulations, namely creams. It was also shown that the natural lipids present in human skin were better candidates for topical application. In this study, liposomes that were composed of saturated phosphatidylcholine performed better in maintaining and penetrating the skin, compared to those composed of unsaturated phosphatidylcholine [ 13 , 56 ].

Singh et al. studied the potential of novel delivery systems, like liposomes and ethosomes entrapping MCZ, against fungal infections. Moreover, they compared implemented liposomes and ethosomes to find the most effective carrier for the administration of antifungal drugs to the skin. It should be noted that they used pigskin in their study. 

Soy lecithin and cholesterol (7:3 ratio) were structural components of liposomes used in the preparation of nanoparticles by the thin-film hydration method. Afterward, ethosomes were prepared by the dropping method in distilled water. The ethosomes included soya lecithin (50 mg/ml) and ethanol (30%). The results of the *in vitro* skin permeation revealed a higher potential of ethosomal MCZ ointment, compared to the liposomal MCZ ointment [ 57 ].

### Terbinafine Hydrochloride

Terbinafine hydrochloride (TBF-HCl), a synthetic allylamine derivative, is a lipophilic antifungal drug administered to treat superficial fungal disease. Its pKa and molecular weight values are 7.1 and 291 Da, respectively. The TBF-HCl has a positive effect on dermatophyte infection as well as an impeding effect on enzyme squalene epoxidase of fungal ergosterol biosynthesis [ 58 ]. 

The therapeutic potential of TBF-HCl liposomes on dermal fungal infections was deeply studied. In a study, liposomes were produced by the ethanol injection method, and their characteristics were examined from different aspects. The results showed that entrapment efficacy was between 39.46% and 70.39%. Regarding size distribution, the maximum and minimum sizes were 344.8 nm and 206.9 nm, respectively. 

Liposomes were loaded to gel and *ex vivo* and *in vitro* studies were conducted on rat skin and artificial membrane, respectively. The results indicated that the liposomal gel with TBF-HCl has excellent efficacy for topical application on the skin, especially for superficial fungal diseases, due to the maximum retention of the drug in the skin. In the above-mentioned study, topical application of liposomes on the skin was strongly recommended as a promising nanocontainer [ 59 ].

TolnaftateTolnaftate (TFT) is an antifungal synthetic thiocarbamate with low solubility. It is commercially available in various topical forms, namely cream, spray, liquid aerosol, and powder [ 60 ]. Topical delivery of TFT incorporated into liposomes was studied. The TFT-loaded liposomes were prepared using the dried thin-film hydration method with different charges. These formulations were entrapped in Carbopol gel, and their different properties and antifungal potentials were examined. 

The sizes of liposomes with different charges varied from 119 nm for neutral liposomes to 284 nm for positive liposomes. Negative liposomes with the size of 143 nm showed better entrapment efficacy which was around 88.14%. Albino Wistar rats were used as the *in vivo* models. It was found that liposomes with smaller vesicle sizes exhibited superior permeation and potential as a treatment for fungal infections. In the aforementioned study, liposomal formulations were suggested as promising topical drug delivery systems to overcome delivery problems [ 61 ].

## Conclusion

There is no doubt that novel drug delivery systems have opened new doors for more effective treatment of diseases. Nanotechnology is a new powerful tool that has provided many facilities and equipment in any field of science, especially medicine and pharmaceutics. Novel nano-drug delivery systems, as fascinating outputs of this new science and technology, have recently emerged and are rapidly growing. 

Different novel carriers have various abilities that must be used in the right place to have the best performance. According to the report of the World Health Organization (Geneva, Switzerland), antimi-crobial resistance is one of the major global problems that threatens human societies. In this case, antifungal drug resistance is of special importance. Usage of extra doses of drugs, non-targeted treatment, and wrong prescription seem to be the main causes of this phenomenon. One way to overcome drug resistance is the application of nanotechnology to deliver drugs. 

As the first drug delivery agent approved by the FDA, liposomes have always been under various investigations, but there are still many hidden aspects to be discovered about them. Amidst the studies on liposomes as antifungal drug carriers to the skin, multiple results are observed concerning their various properties, components, and types of drugs. Liposomes are safe, biocompatible, biodegradable, and able to deliver both hydrophobic and hydrophilic molecules. Besides, liposomes can act as controlled release systems and are known as versatile, targeted delivery systems. 

According to the literature review summarized in this paper, almost all of the studies have identified liposome as a powerful tool in topical delivery to the skin. However, a few studies have introduced new generations of liposomes, like ethosomes and transfersomes, and compared them with liposomes that demonstrated controversial results. This reliable activity of liposomes seems to be due to the active site of most superficial fungal infections in the epidermis, which is also the depot site of liposomes as topical carriers to the skin.

Based on the available information about liposomes as topical carriers to the skin to date and according to the results of the studies conducted about the topical delivery of antifungal agents against superficial fungal infections, topical liposomes seem to be promising versatile carriers for the treatment of superficial fungal diseases.

## Authors’ contribution

P. A. and N. S. wrote the manuscript, A. M. edited the manuscript, and M. A. was a scientific adviser in the field of mycology. Furthermore, J. A. participated in designing the study, searching for proper papers, writing the manuscript, and designing the figures. 

## Financial disclosure

This study was supported by a grant from Mazandaran University of Medical Sciences, (Grant Number: 8503) with the ethical code of IR.MAZUMS.REC.1399.8503.
